# The Regulation of the Hippo Pathway by Intercellular Junction Proteins

**DOI:** 10.3390/life12111792

**Published:** 2022-11-05

**Authors:** Usama Sharif Ahmad, Jutamas Uttagomol, Hong Wan

**Affiliations:** 1 Centre for Oral Immunobiology and Regenerative Medicine, Institute of Dentistry, Barts and The London School of Medicine and Dentistry, Queen Mary University of London, London E1 2AT, UK; 2 Oral Diagnosis Department, Faculty of Dentistry, Naresuan University, Phitsanulok 65000, Thailand

**Keywords:** Hippo pathway, YAP, TAZ, phosphorylated YAP, cell–cell junctions, adherens junctions, tight junctions, desmosomes, desmoglein-3

## Abstract

The Hippo pathway is an evolutionarily conserved pathway that serves to promote cell death and differentiation while inhibiting cellular proliferation across species. The downstream effectors of this pathway, yes-associated protein (YAP) and transcriptional co-activator with PDZ-binding motif (TAZ), are considered vital in promoting the output of the Hippo pathway, with activation of upstream kinases negatively regulating YAP/TAZ activity. The upstream regulation of the Hippo pathway is not entirely understood on a molecular level. However, several studies have shown that numerous cellular and non-cellular mechanisms such as cell polarity, contact inhibition, soluble factors, mechanical forces, and metabolism can convey external stimuli to the intracellular kinase cascade, promoting the activation of key components of the Hippo pathway and therefore regulating the subcellular localisation and protein activity of YAP/TAZ. This review will summarise what we have learnt about the role of intercellular junction-associated proteins in the activation of this pathway, including adherens junctions and tight junctions, and in particular our latest findings about the desmosomal components, including desmoglein-3 (DSG3), in the regulation of YAP signalling, phosphorylation, and subcellular translocation.

## 1. Introduction

The Hippo pathway is an evolutionarily conserved pathway that controls tissue growth and organ size. It was first discovered during the 1990s in *Drosophila melanogaster* as a result of genetic mosaic screens for tumour suppressor genes and genes associated with organogenesis and development [1,2]. Inactivation of several genes in this pathway in *D. melanogaster* leads to the same phenotype where tissue overgrowth is pronounced, suggesting disruption of normal controls of cell growth and proliferation [1,3,4,5]. The Hippo (*Hpo*) protein gets its name from the phenotype observed when it is mutated, producing a much larger tissue mass than usual due to excessive proliferation. The researchers that made this first observation matched the appearance of *D. melanogaster* to that of a hippopotamus due to the thickened and wrinkled skin [6]. This unusual phenotype occurs due to the inability of Hippo to carry out its normal cellular functions, which include growth attenuation via apoptosis [7]. The downstream effectors of the Hippo pathway are the yes-associated protein (YAP) and its paralog transcriptional co-activator with PDZ-binding motif (TAZ), with their activities being regulated by post-translational phosphorylation that inactivates YAP/TAZ, leading to their cytoplasmic translocation and/or degradation. 

The Hippo pathway was noted to be an essential regulator of organ size, development, and homeostasis in the organism [8,9]. When various cell types are grown in culture, they eventually reach confluence and stop growing. Similarly, in the body, when they grow to occupy a given space, cells then stop growing upon contact with each other. Given that cells grow and divide, how do they know when to stop? How do they know when the organ they are building is the correct size? This contact inhibition has been recognised for decades but only has been identified recently as being partly due to the Hippo pathway. The initial screening process for genes involved in the Hippo pathway was focused on an appreciation of growth control mechanisms for organ development, the processes that are poorly defined in genetic terms [10]. Key components of the Hippo pathway were identified during these genetic screens. This review aims to provide an overview of the significance of this pathway in *D. melanogaster* and mammals, with a focus on its regulation by intercellular junctional components.

## 2. Methods

The literature search was carried out in the NCBI PubMed database using keywords “Hippo pathway”, “yes-associated protein”, “YAP”, “tight junctions”, “adherens junctions”, and “desmosomes”. 

## 3. The Hippo Pathway

### 3.1. Biological Functions of the Hippo Pathway 

The initial analysis for the genes involved in this pathway included Warts *(Wts),* Hippo *(Hpo),* Salvador *(Sav),* and Mats *(Mats)* as tumour suppressors, which shared similar functions that regulate aspects of cell growth and tissue size control [7,11,12]. Indeed, the same genes were associated with phenotypes of an overgrowth of imaginal discs and corresponding structures in adult fruit flies. Such defects are the result of hyperproliferation of cells and a lack of cellular regulation by apoptosis (programmed cell death); and an imbalance of cellular growth, proliferation signalling, and regulated cell death, leading to aberrant organ development [3]. The functions of the Hippo pathway are diverse and reflect cellular regulatory events that control tissue growth and development. Primarily, the pathway serves to promote cell death and differentiation while inhibiting cellular proliferation across species [13]. The initial findings in *D. melanogaster* were replicated in mice models following the identification of homologous genes [14]. This finding not only illustrates the highly conserved nature of the Hippo pathway across species but also emphasises the consistent effects of Hippo pathway mutations on growth and development [15]. Furthermore, the homologous genes for *Hpo* in mice were identified as *Mst1* and *Mst2*, and it was found that deletion of these genes led to the proliferation of hepatocytes during liver development, resulting in hepatomegaly [16]. Consequently, these experiments have further solidified the possibility of the Hippo pathway as a regulator of organ growth in mice and fruit flies.

Elucidation of the key components of the Hippo pathway has led to an expanded knowledge of the mechanisms involved in cell growth and organogenesis while highlighting the putative roles of individual proteins in this pathway. The components of the Hippo pathway are considered in greater detail in the following section.

### 3.2. Components of the Hippo Pathway and Associated Functions

The Hippo pathway comprises several proteins, including kinases, downstream effectors, and transcription factors. This pathway can be divided into two parts, namely, a cytoplasmic component that comprises a serine/threonine kinase cascade that is activated from upstream cues, which are considered tumour suppressors, and nuclear transcription module components that are downstream effectors of this pathway, which act as oncogenes [17]. The core components of the Hippo pathway are the serine/threonine kinases involved in a signalling cascade; a list of known proteins involved in the pathway (in *D. melanogaster* and humans) is summarised in Table 1. There are over 30 components identified in the Hippo pathway, suggesting a complex signalling cascade, while specific genes and their products appear to have key functions within the pathway.

The roles of upstream kinases within the Hippo pathway are diverse, and the regulation of these kinases has profound effects on the pathway’s activation. Furthermore, the downstream targets involved in producing the physiological/genetic effects of the pathway are equally diverse [18]. Some key molecules within the pathway warrant further discussion based on the strength of their effect in the context of cell growth and development and potentially in tumour contexts, reflecting their importance in the pathway.

The mammalian Hippo kinase cascade is initiated by serine/threonine protein kinases (TAOK1/2/3). However, two main kinases regulate the activity of the downstream targets: mammalian STE20-like kinase 1/2 (MST1/2), and large tumour suppressor kinase 1/2 (LATS1/2), the orthologues of *D. melanogaster* Hippo and Warts, respectively [19]. Both kinases serve to inhibit the primary effectors of the Hippo pathway, and thus their regulatory control has an influence on the effects of the pathway on growth and development and broader homeostasis [20]. 

Activation of TAOK1/2/3 leads to phosphorylation and the activation loop of MST1 at Thr183 and MST2 at Thr180, thereby leading to MST1/2 activation [21]. MST1/2 can also undergo auto-activation through MST1/2 autophosphorylation by dimerization [22]. The active MST1/2 phosphorylates the adaptor proteins Salvador family WW domain-containing protein 1 (SAV1) and MOB kinase activator 1A/B (MOB1A/B) [23]. Both scaffold proteins play a crucial role in recruitment and in facilitating interactions between MST1/2 and LATS1/2. MOB1A/B forms a complex with LATS1/2, allowing MST1/2 to phosphorylate LATS1 at Thr1079 and LATS2 at Thr1041, which subsequently leads to the activation of LATS1/2. In addition, phosphorylation of MOB1A/B leads to its affinity and interaction with LATS1/2, causing a cascade of autophosphorylation of this protein within the activation loop [24,25]. The autophosphorylation of LATS1/2 and by MST1/2 are required for LATS1/2 kinase activity. The phosphorylation and activation of LATS1/2 can also occur independently of MST1/2. For example, TAOK1/3 can directly phosphorylate and activate LATS1/2 at its hydrophobic motif [10]. In addition, MAP4K4/6/7 and MAP4K1/2/3/5 can also phosphorylate and activate LATS1/2 in parallel with MST1/2 [20,26]. Once LATS1/2 is activated, it can directly phosphorylate orthologues of the *D. melanogaster Yki* gene product—YAP and TAZ [27]. 

The nuclear transcription co-effector YAP/TAZ proteins are considered vital in promoting the output of the Hippo pathway, with Hippo pathway activation negatively regulating (inhibiting) YAP/TAZ activity [28]. Phosphorylation of YAP at serine-127 by LATS1/2 leads to sequestration of YAP in the cytoplasm, with binding to protein 14-3-3 or subsequent degradation of the protein [13]. Similarly, the activity of LATS1/2 phosphorylates TAZ and affects the localisation of this protein, reducing its stability within the cytoplasm [29]. When LATS1/2 are inactive, YAP/TAZ remain unphosphorylated; they translocate to the cell nucleus, acting as transcription co-activators, binding to the transcriptional enhanced associate domain (TEAD) transcription factor family (TEAD1-4) (*Sd* orthologs). Inside the nucleus, the YAP/TAZ-TEAD protein complex promotes the transcription of a wide range of genes related to cell growth, proliferation, migration, apoptosis, and other homeostatic mechanisms associated with survival [30,31,32,33].

**Figure 1:**
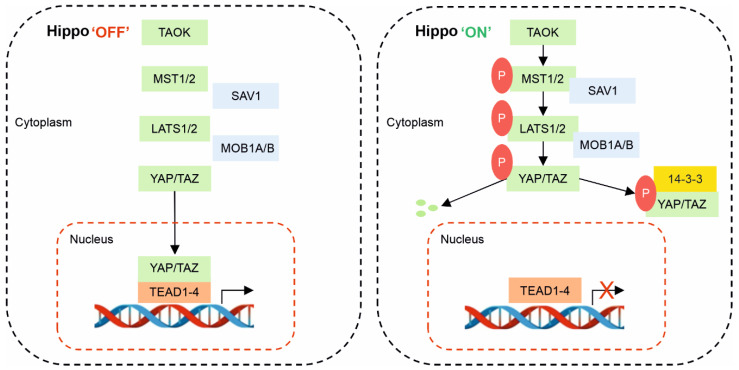
The Hippo pathway in mammals. When the pathway is inactive (Hippo “OFF”), YAP/TAZ remain unphosphorylated and are localised to the nucleus, where they bind to TEAD and activate gene transcription. When the pathway is active (Hippo “ON”), upstream stimuli phosphorylate and activate TAOK kinases, which phosphorylate MST1/2. MST1/2 then, in turn, phosphorylates LATS1/2, which is facilitated by adaptor proteins SAV1 and MOB1A/B. Finally, the activated LATS1/2 phosphorylates YAP/TAZ, leading to YAP/TAZ ubiquitination and proteolytic degradation or allowing their interaction with protein 14-3-3 and cytoplasmic retention, rendering them unable to maintain their action in the nucleus.

As summarised in Figure 1, the basics are simply that while YAP and TAZ remain in the nucleus in their unphosphorylated states (Hippo off), they regulate the expression of genes associated with cellular growth, proliferation, development, migration, and survival. However, when inactivated via phosphorylation (Hippo on), YAP and TAZ are translocated and retained in the cytoplasm, where they are either degraded or bind to protein 14-3-3, reducing cellular activity and potentially triggering apoptosis under certain conditions [34].

The loss of the core components (MST1/2, SAV1, MOB1A/B, and LATS1/2) has been shown to lead to the upregulation of YAP/TAZ-TEAD target gene expression [35,36]. Gene deletion of loss-of-function studies in *D. melanogaster* provides support for the importance of these components in the Hippo pathway, where uncontrolled growth is seen following the loss of the inhibitory effects of the Hippo pathway on Yorkie, the orthologues of mammalian YAP/TAZ [32]. Gene deletion studies in mice have also supported this finding, based on observations of uncontrolled tissue growth and organomegaly following the deletion of proteins that would inhibit YAP/TAZ activity [37]. Therefore, the regulation of the Hippo pathway, including the YAP/TAZ proteins, appears to have significance in the context of development, particularly in diseases such as cancer, where poorly regulated cell growth is a hallmark of the condition.

## 4. Regulation of the Hippo Pathway by Intercellular Junction Proteins

**Figure 2:**
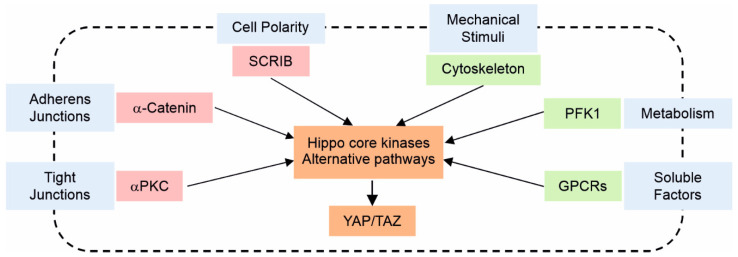
The regulation of the Hippo pathway. Many upstream regulators of the Hippo pathway have been identified, including cell polarity, cell–cell adhesion, mechanical stimuli, metabolism, and soluble factors. The activation of signalling pathways leads to YAP/TAZ activity regulation through the Hippo core kinases or alternative pathways. Examples of multiple inputs are shown in blue; pathways that inhibit YAP are shown in red, and pathways that activate YAP are shown in green.

Upstream regulation of the Hippo pathway is not entirely understood on a molecular level. However, several studies have shown that extracellular, intracellular, and non-cellular mechanisms can activate Hippo signalling and therefore regulate the subcellular localisation and protein activity of YAP/TAZ through phosphorylation of upstream kinases. Some of these mechanisms include intracellular junctions, cell polarity, mechanical stimuli, and contact inhibition (Figure 2). In addition, these mechanisms convey external stimuli to the intracellular kinase cascade, promoting the activation of key components of the Hippo pathway [38]. Here in this review, we focus on some of these mechanisms involved in cell junction components that have been identified in the regulation of this pathway in the literature. For others, please refer to reviews [34,39,40,41]. 

There is much evidence linking the role of intracellular junctions and the activation of the Hippo pathway in multiple cell types [42]. Initial findings that cell–cell junctions regulated the Hippo pathway were based on observations that the neurofibromatosis type 2 (NF2) and Merlin (Mer) proteins are required for Hippo signalling to occur [43] (discussed below). The Expanded (Ex) FERM-domain adaptor protein may also play a role in the initial pathway activation [44]. NF2/Mer and Ex complexes have been shown to localise to tight and adherens junctions within epithelial cells, and the Mer protein is specifically necessary for the formation of functional adherens junctions [42]. The NF2/Mer proteins also play a key role in the activation of YAP and TAZ via intermediary proteins [13,45]. These observations led to a wide range of investigations that identified several associations between Hippo pathway components and tight or adherens junctions between cells.

### 4.1. Regulation of YAP/TAZ by Tight Junctions

Hippo pathway components are not regulated by a single cellular receptor or external stimuli. Instead, they appear to have a wide range of potential effectors and activators, particularly given their important association with cellular junctions and mechanisms of protein activation associated with junction assembly [46]. Tight junctions (TJs) form apical barriers to the permeability of small molecules and proteins between epithelial sheets and play a role in the Hippo pathway. Many upstream effectors of the Hippo pathway are associated with TJ protein complexes, including Crumbs and atypical protein kinase C (aPKC) [47,48]. Several components of TJs and the integral parts of TJs are involved in the regulation of Hippo pathway.

#### 4.1.1. Claudins

Claudins are a family of TJ integral membrane proteins that are key regulators of the paracellular permeability and cell polarity [49,50]. Various members of claudin family are reported to associate with the Hippo pathway regulation. CLDN18 is one of the claudin family members which is highly expressed in lung alveolar epithelium [51]. It is reported to interact and colocalise with YAP at cell–cell contact. Overexpression of CLDN18 reduced YAP nuclear localisation, cell proliferation, and YAP transcriptional activity and the loss of CLDN18 suppresses the interaction of YAP and Hippo kinases phopsho-LATS1/2 in alveolar epithelial cells [52]. These results have identified the potentially negative regulator of CLDN18 to YAP activity. 

Claudin-2 (CLDN2) has recently been demonstrated to interact with proteins that regulate cell-cell adhesion and cell polarity [53,54]. It is reported that CLDN2 co-immunoprecipitated with YAP and a role of YAP-activation in renal clear cell carcinoma has been established [55,56,57]. Kumar and colleagues revealed that CLDN2 associates with YAP protein through its PDZ binding motif and is sequestered to the membrane in differentiated proximal tubular epithelial cells. Downregulation of CLDN2 expression, due to the induction of cancer promoting factors, promotes loss of cell–cell contact and YAP translocation to cell nucleus leading to the proliferation and the progression of renal clear cell carcinoma [56]. CLDN2 also activates YAP in human colorectal cancer stem-like cells resulting in self-renewal of cells [58].

Claudin-6 (CLDN6) has been found to be highly upregulated both at the mRNA and protein levels in gastric cancer cell lines and tissues indicating poor prognosis [59]. CLDN6 interacts with LATS1/2 and leads to the reduction of LATS1/2 and YAP phosphorylation, thereby stimulating YAP nuclear accumulation and activating downstream target genes [59]. Nuclear claudin-4 (CLDN4) expression showed a stronger relation with cancer progression than the nuclear negative immunoreactivity in oral squamous cell carcinoma (OSCC). Treatment of human OSCC cell lines with clostridium perfringens enterotoxin (CPE), the anaerobic bacteria found in the plaque-associated bacterial flora in the oral cavity, promotes CLDN4 nuclear translocation, impairs TJs, activates YAP, and enhances the epithelial–mesenchymal transition (EMT), cell proliferation, and invasion capability [60]. Similar effects are also evident in colonic sessile serrated adenoma/polyps with dysplasia [61] as well as in colorectal cancer [62]. In addition, CPE treatment induces the formation of YAP1-CLDN4-zona occludens-2, which in turn decreases YAP1 phosphorylation by LATS and activates YAP1. The addition of CLDN4 in this complex may mask the phosphorylation site of YAP1 [60]. These findings revealed the interaction between CLDN4 and YAP.

#### 4.1.2. Zonula Occludens

Zonula occludens proteins (ZO-1, ZO-2, and ZO-3) belong to the family of membrane-associated guanylate kinase homologue proteins providing the structural basis for multiprotein complex assembly at the cytoplasmic surface of intercellular junctions [63,64]. ZO proteins can interact with proteins localising to the nucleus and the plasma membrane [63]. ZO-1 colocalises with YAP at the cell membrane and knockdown of ZO-1 exhibits an increase of nuclear YAP and a reduction of YAP at cell junctions. The association of YAP and ZO-1 is mediated by angiomotin (AMOT) in gastric cancer cells [65]. ZO-2 binds to YAP through YAP’s PDZ-binding motif, which can lead to the nuclear accumulation of YAP at low cell densities [66,67]. On the other hand, ZO-2 is also reported to be required for efficient phosphorylation and the relocation of YAP from the nucleus to the cytoplasm and also stimulates the formation of a tripartite complex of LATS1-ZO-2-YAP in the cytoplasm at high cell densities [68,69]. The tripartite complex is then recruited to TJs, where LATS1 is already activated by AMOT and NF2, resulting in the effective phosphorylation of YAP (inactivation of YAP in the cytoplasm) [69]. ZO-2, like YAP, predominantly accumulates in the nucleus in sparse cell density conditions. ZO-2 silencing reduces YAP phosphorylation at the S127 residue [69], triggers the accumulation of YAP in the nucleus, and promotes its transcriptional activity, which, in turn, results in an increase in cell growth [68]. Thus, ZO-2 may play a novel role as a cell size modulator [68] which utilises the cell–cell cohesion status to control the phosphorylation status of YAP [68,69].

#### 4.1.3. Angiomotin Family

The AMOT family of proteins (which associate tight junctions to the internal cytoskeleton) directly binds and negatively regulates YAP activity and localisation both directly and indirectly of Hippo signalling [46,70]. AMOT proteins interact with multiple TJ components and are essential for maintaining TJ integrity and epithelial cell polarity [71]. The AMOTs protein family consists of AMOT, AMOTL1, and AMOTL2 [72]. The interaction between AMOTs and YAP/TAZ has been identified and is mediated through direct protein–protein interaction between the AMOT PPxY motifs and the YAP/TAZ WW domains. The interaction can also occur independently of the phosphorylation status of YAP/TAZ [67,73,74,75]. Zhao and colleagues reported that AMOTs recruit YAP/TAZ to TJs or the actin cytoskeleton, leading to a reduction of YAP/TAZ nuclear localisation and activity. AMOTs also induce YAP/TAZ phosphorylation at the LATS target sites [75]. Thus, AMOTs can inhibit YAP/TAZ activity by both phosphorylation-dependent and -independent mechanisms. 

AMOTL2 interacts with LATS2 and YAP at TJs, which can promote LATS2-mediated YAP phosphorylation, thereby causing the inactivation of YAP [76]. AMOTL2 depletion in non-transformed mammary epithelial cells (MCF10A) results in enhanced nuclear localization of YAP and EMT [74]. AMOTL1 forms a tripartite complex with ZO-2 and YAP and regulates YAP function in opposite directions. AMOTL1 prevents pro-apoptotic functions of YAP, while ZO-2 does the opposite [67]. 

#### 4.1.4. Crumbs Complex

The Crumbs (Crb) complex is a key modulator of cell shape and is essential for apical–basal cell polarity of epithelial cells, which is crucial for organising cellular components and determining a cell’s relative position in a tissue or local microenvironment while governing aspects of cell activity transcriptional gene regulation in response to external stimuli [77]. Crb3, a Crumb isoform that determines epithelial apical domain identity [78], is expressed preferentially in epithelial tissues and skeletal muscles [79]. Crb3 has been shown to stimulate the interaction between YAP and LATS1/2 at apical cell junctions by inducing YAP phosphorylation and cytoplasmic retention, which consequently drives airway epithelial cell differentiation [78]. Loss of Crb3 in high cell-density cultures strongly suppresses YAP phosphorylation and increases nuclear YAP/TAZ [80], and a similar effect occurs in Schwann cells [81]. Furthermore, other studies have shown that Crumbs, in association with proteins FERM domain-containing protein 6, Kibra, and Merlin, can activate the Hippo pathway and inhibit YAP activity [82,83]. 

#### 4.1.5. Atypical Protein Kinase C

The atypical protein kinase C (aPKC) complex consists of the Par3 and Par6 adaptor proteins and the aPKC serine–threonine kinase [42,84]. This complex is the main cell polarity regulatory engine in all eukaryotes [84]. It is reported that disruption of the aPKC complex induces Hippo signalling and decreases YAP nuclear localisation in the outer cells of 16-cell and 32-cell stage mouse embryos [85]. In addition, the overexpression of the aPKC complex in human cancers negatively regulates the Hippo pathway, resulting in the nuclear accumulation of YAP in epithelial cells [86]. Taken together, TJ components have been strongly linked to Hippo pathway activation and the subsequent control of growth, reflecting the importance of mechanical stresses and external stimuli on the internal growth of cells [46]. 

### 4.2. Regulation of YAP/TAZ by Adherens Junctions

In addition to tight junctions, adherens junctions (AJs) between epithelial cells are also associated with Hippo pathway regulation and have been shown to stimulate the Hippo signalling pathway and inhibit cell growth [87,88].

#### 4.2.1. E-Cadherin and α/β-Catenin

In keratinocytes, cadherins are a critical component that bind cells with each other [89]. These junctions mediate adhesion between epithelial cells via E-cadherin that forms an apical band around the cell and complexes with catenins (e.g., α-catenin and β-catenin) in the cytoplasmic domain that connects cadherins to the actin cytoskeleton. These junctions are crucial for maintaining tissue architecture and the relationship between cells in the epithelium, while cadherins and catenins also provide molecular links to the Hippo pathway [87].

α-Catenin acts as a potent inhibitor of YAP activity, and such an inhibition of YAP may contribute to its tumour suppressor functions [90,91]. α-Catenin can suppress YAP activity by forming a complex with 14-3-3 proteins and phosphorylated YAP, causing YAP inactivation by retaining YAP in the cytoplasm [90]. The trimeric complex of α-catenin, 14-3-3, and YAP sequesters YAP at AJs and prevents YAP activation in the nucleus. Knockdown of α-catenin has been found to induce YAP/TAZ nuclear localisation [80]. In mammalian cells, cell–cell adhesion mediated by homophilic binding of E-cadherin, independent of other cell interactions, is sufficient to cause YAP inactivation [87]. Disturbing the E-cadherin/α-catenin complex results in a decrease in YAP phosphorylation and an increase in YAP nuclear accumulation and activity [87,92]. β-Catenin forms a complex with nuclear YAP, which is essential for cardiac development [93]. Concurrent nuclear localisation of β-catenin and YAP is also evident in human hepatoblastoma. Knockdown of YAP or β-catenin decreases proliferation in hepatoblastoma cells [94]. Evidence from disease studies also supports an association between adherens junctions and the Hippo pathway, as loss of catenins or cadherins is commonly seen in epithelial cell cancers. In contrast, the expression of dysfunctional forms of cadherins (e.g., β-catenin) is associated with cancer and has been shown to modify YAP translocation in the cell, leading to nuclear localisation and cellular growth signalling [42].

#### 4.2.2. Protein Tyrosine Phosphatase Non-Receptor Type 14 (PTPN14)

PTPN14 is one of six protein tyrosine phosphatases that are involved in protein tyrosine phosphorylation [95]. It was initially identified as a cytoskeleton-associated phosphatase involved in cell proliferation and adhesion [96]. PTPN14 has been found to mediate β-catenin dephosphorylation at AJs, which is important for E-cadherin/β-catenin linkage and cell adhesion [97]. PTPN14 can directly interact with YAP, causing YAP cytoplasmic retention and inactivation [98]. It forms a protein complex with the WW domains of YAP through its PPXY motifs, inhibiting YAP-mediated transcriptional activities [99,100]. It is thought that regulation of Hippo pathway kinases and sequestration of YAP occurs at the site of adherens junctions based on the localisation of some Hippo components at these junctions [101]. Knockdown of PTPN14 has been shown to induce YAP nuclear retention and increase YAP-dependent cell migration [100]. Wang and colleagues revealed that PTPN14 can translocate YAP from the nucleus to the cytoplasm via their physical interaction [98]. Furthermore, PTPN14 also interacts with the Kibra protein through the PPXY domain of PTPN14 and the WW domain of Kibra. Their interaction can induce LATS1 activation, leading to the subsequent cytoplasmic sequestration of YAP [102]. Therefore, these findings support that PTPN14 functions as a positive regulator of the Hippo pathway and inactivates YAP.

#### 4.2.3. Merlin

Mer, an important inhibitor of YAP/TAZ, is encoded by the neurofibromatosis type 2 (NF2) tumour suppressor locus [103]. Mer is related to the ezrin, radixin, and myosin (ERM) family of membrane cytoskeleton-associated proteins [104]. Mer is localised in close proximity to AJs and TJs in the confluence of mammalian epithelial cells, and this location seems to be essential for Mer-mediated tumour suppression [104,105,106]. At cell–cell junctions, Mer may enhance assembly of the proper protein scaffolds, which enable LATS activation and YAP phosphorylation. Kibra (Kbr, a WW and C2 domain-containing protein) was identified to function together with Mer and colocalises at the apical domain of epithelial cells. Kibra is part of an apical scaffold that promotes Hippo pathway activity. It may serve as a bridge between Mer and LATS [83,107,108,109]. Ying and colleagues found that Mer directly binds and recruits LATS to the plasma membrane, in promoting phosphorylation of Warts by the Hippo–Sav kinase complex [110]. A study in 2010 demonstrated that the Mer/NF2 tumour suppressor and the YAP oncoprotein function antagonistically to regulate liver development [111]. Inactivation of NF2 resulted in hepatocellular carcinoma and bile duct hamartoma, whereas inactivation of YAP led to the loss of hepatocytes and biliary epithelial cells. Overexpression of NF2 in mammalian cells led to LATS activation and YAP inhibition [111]. These results suggest that Mer/NF2 is an upstream component of the mammalian Hippo pathway in the control of cell proliferation.

#### 4.2.4. Ajuba

Mammalian ajuba family members such as AJUBA, LIMD1, and WTIP are associated with α-catenin at AJs [42]. They are recruited to AJs in a tension-dependent manner by α-catenin [112,113,114]. Ajuba family members demonstrate their ability to regulate the Hippo signalling pathway, including YAP/TAZ functions [115]. Their binding to LATS1/2 reduces the activity of the Hippo pathway [116,117]. The interaction between ajuba family proteins and LATS kinases can be stimulated by JNK or ERK phosphorylation, which thereby induces nuclear localisation and the transcriptional activity of YAP [118,119]. However, ajuba family proteins have been found to inhibit Hippo regulation of YAP only in proliferating cells, whereas they seem to not suppress or associate with the Hippo kinase complex in growth-arrested cells in vitro [120].

In the reconstituted epidermis, YAP, and WW-binding protein 2 (WBP2), a YAP co-factor enhancing YAP/TEAD-mediated gene transcription, are regulated by intercellular adhesion rather than canonical Hippo signalling. Impairment of the intercellular adhesion promotes nuclear YAP/WBP2 binding, which leads to the outgrowth of cutaneous squamous cell carcinoma [121]. Disruption of AJs or TJs by EMT or the other regulation mechanisms also induces YAP/TAZ activity. Hence, disturbing the cell’s adhesive properties may inhibit the Hippo cascade indirectly via the mechanism in the loss of cell cytoarchitecture and cell polarisation [28]. These results indicate that apical–basal polarity, cell–cell contact, and the integrity of cell junctions all are important in regulating the Hippo pathway [41].

### 4.3. Regulation of YAP/TAZ by Cell Polarity

Cellular polarity and architecture are particularly crucial in the growth of epithelial cells, where the definition of the apical–basolateral polarity of the cell is crucial to preventing dysplasia [42]. Loss of cellular polarity is an initial step in the epithelium to mesenchymal transition as part of the oncogenic process, whereby epithelial cells adopt a fibroblast-like morphology, consistent with the migratory and metastatic potential of cancer cells. Three main cellular polarity components are considered relevant to the Hippo pathway: apically located Crumbs, basally located Scribble (SCRIB) complexes, and cadherin–catenin complexes at AJs at apical and basolateral membranes [122]. The Crumbs complex at the apical membrane contains the AMOT protein, which recruits kinases in the Hippo pathway, leading to the phosphorylation of YAP by LATS1/2 [123]. Accordingly, this mechanism prevents YAP entry/translocation to the cell nucleus and subsequent transcriptional events [27]. Similarly, Scribble is known as a key gatekeeper of the epithelial polarity and localises at the basolateral domain of the cell membrane [124]. Scribble complexes form a membrane-localised complex with members of the Hippo pathway, leading to the phosphorylation of TAZ and subsequent degradation of the protein [125]. EMT causes Scribble delocalisation and inactivates the Hippo cascade, which consequently leads to TAZ (and likely YAP) activation with the acquisition of cancer stem cell-like traits [126]. In epithelial cancers (e.g., SCCs), Scribble activity is noted to be weak or null, leading to a loss of YAP/TAZ phosphorylation and degradation [127].

### 4.4. Regulation of YAP/TAZ by Mechanotransduction

Cell contact/density and mechanical stimulation such as strain and stress are essential aspects of the Hippo activation and regulation process, stemming from anatomical considerations of organ growth in a finite body space, where physical restraints and cellular mechanical cues are necessary [88,125,128]. During organogenesis, tissue architecture is essential in restricting cell growth and ensuring cellular quiescence to prevent anatomical abnormalities or disease [129]. In high-density contexts, cell-to-cell contact can result in the generation of inhibitory signals that serve to inhibit growth; it is thought that such signals are primarily mediated through the Hippo pathway [130]. Furthermore, cell contact inhibition in cell cultures leads to YAP inactivation. Cell-to-cell contact results in LATS1/2 kinase activity, leading to YAP inactivation at a high cell density, whereas at a low cell density, LATS1/2 is inactive. The increased TJs and AJs in confluent cultures bring about LATS1/2 activation and subsequent inactivation of YAP/TAZ [27,96].

YAP/TAZ are regulated by cell geometry. Nuclear YAP/TAZ activity is seen in cells undergoing spreading, whereas inactivation of YAP/TAZ is seen in the round and compact cells [130]. Indeed, mechanical stimulation in the form of modifications in cytoskeletal dynamics or the action of the extracellular matrix proteins has been shown to modify the transcriptional activity and cellular location of Hippo pathway components [131]. YAP/TAZ are located in the nucleus and are transcriptionally active in a dense medium, while in a highly dense context where mechanical stimuli from the substrate are weak, YAP/TAZ are localised to the cell membrane or cytoplasm and are inactive [132]. The reorganisation of the actin cytoskeleton, such as the F-actin protein and the activity of Rho-GTPases in response to mechanical stimuli, has been shown to activate YAP/TAZ activity in fruit flies and mammals [133].

Moreover, cells’ physical attachment and detachment to the extracellular matrix (ECM) can induce or repress YAP/TAZ activity. When cells attach to the ECM, YAP is localised to the nucleus through the FAK-Src-PI3K pathway or the activation of Rho-GTPases. On the other hand, the detachment of cells by anoikis results in the inhibition of YAP/TAZ [134]. In addition, focal adhesions (FA) connect the cytoskeleton of cells to the ECM. Upon activation through mechanical stimulation or ligands, integrins in FA have been shown to activate YAP/TAZ through Rho-GTPases and also through Src kinase signalling [135].

### 4.5. Regulation of YAP/TAZ by Desmosomes

Desmosomes (DSM) are another type of intercellular anchoring junction essential for the maintenance of the integrity of tissues such as skin, myocardium, and gastrointestinal mucosa that experience mechanical stresses. Rather than the actin cytoskeleton, DSMs tether the intermediate filaments to the plasma membrane and adhere the adjacent cells by the desmosomal cadherins, thus forming a structural network known as the desmosome–intermediate filament complex (DIFC) that is essential to maintain the architecture of epithelial tissues [136]. There are very limited studies in the literature investigating the involvement of desmosomal proteins in regulating Hippo signalling.

Lack of plakophilin-2 (PKP2) leads to YAP phosphorylation through the activation of LATS1/2 kinases in cardiomyocytes. Desmoplakin (DSP) and plakoglobin (PG) depletion has been found to activate the phosphorylation of YAP, predominantly at the cell membrane. Furthermore, co-immunoprecipitation-detected binding of PG and YAP is also evident. These results from cardiomyocytes indicate that these desmosomal proteins are involved in the activation and localisation of Hippo pathway components [137]. The next section explores the role of desmoglein-3 (DSG3) in the regulation of Hippo signalling, and the recent findings reported in the literature.

## 5. DSG3 in the Regulation of Hippo-YAP Signalling

DSG3 belongs to the cadherin superfamily and plays a vital role in cell junction formation in epithelial cells. In addition to its function in desmosome cell–cell junctions, DSG3 expression has been noted to be distributed along the entire cell membrane, suggesting that DSG3 is involved in non-junctional cellular events [138]. Considering this, DSG3 is the primary target autoantigen in pemphigus vulgaris (PV), a blistering autoimmune disease of the skin and oral mucous membrane [139]. The circulating autoantibodies targeting DSG3 cause disruption of cell cohesion and the loss of DSG3 from the keratinocyte surface. Likewise, DSG3 also acts as a cell signal regulator in several cellular signalling pathways (Src, Rac1/cdc42, Ezrin, c-Jun, and Rho GTPases) involving proliferation, membrane trafficking, migration, and invasion [140,141,142,143,144,145,146]. Overall, the accumulating evidence strengthens the DSG3’s notion as a signalling protein. Additionally, since Hippo pathway components, including YAP, temporarily localise to junctional complexes, desmosomes such as DSG3 could potentially regulate Hippo signalling [147].

YAP is known as a microenvironment sensor, responding to structural and mechanical cues [130,131,148,149,150,151]. In comparison, our recent finding sheds light on the mechanosensing and mechanotransduction roles of DSG3 in response to mechanical forces (i.e., cyclic strain and substrate stiffness) that have an impact on YAP phosphorylation and subcellular translocation, supporting the notion that DSG3 may regulate YAP via recruiting phospho-YAP-S127 (p-YAP) nuclear export [152]. Using HaCaT cells (a human skin keratinocyte cell line), we found that DSG3 depletion resulted in a reduction of YAP and p-YAP expression, which led to the downregulation of *YAP1* target genes associated with cell proliferation. Importantly, we found that DSG3 colocalises and forms a complex with p-YAP and sequesters it to the plasma membrane [152]. Together, these results indicate that DSG3 is capable of regulating the expression and localisation of YAP.

Moreover, another study by our group reported elevated YAP protein levels in the oral mucosa of patients with PV (in which DSG3 is disrupted by autoantibody targeting), and antioxidant treatment of keratinocytes exposed to PV sera suppressed nuclear YAP accumulation and enhanced DSG3-mediated junction assembly [153]. Moreover, we showed that overexpression of YAP disturbs intercellular junction assembly, and YAP knockdown resulted in an inverse effect with increased expression of junction assembly proteins such as α-catenin and DSG3, implying that DSG3 can be negatively regulated by YAP [153].

In support of this, a further study by our group showed that YAP knockdown in OSCC cell lines H157 and H413 led to enhanced expression of DSG3 at the gene and protein levels with inhibition of cell migration [154]. This finding agreed with a recent report in our lab based on skin keratinocytes [153], suggesting negative regulation between DSG3 and YAP. Furthermore, YAP knockdown led to elevated expression and membrane localisation of AJ proteins E-cadherin/α-catenin and desmosomal plaque proteins PKP1/3 [154]. Our results confirm the reciprocal negative correlation between YAP and cell–cell junctional proteins that is in line with Hippo signalling. E-cadherin/catenin-mediated complex formation during junction formation, which also involves DSG3 [145,146], has been shown to act as an upstream regulator of the Hippo pathway leading to YAP nuclear exclusion and inactivation [87,90,152].

Furthermore, in this study we also showed that DSG3 overexpression in OSCC H413 cells resulted in a marked inhibition of cell migration in both the collective and colony settings. These results phenocopied with YAP knockdown cells that exhibited upregulation of DSG3 [154]. To gain a mechanistic understanding of how DSG3 regulates YAP, we demonstrated that DSG3 inhibits YAP activity by inducing phospho-YAP expression. This was showed by Western blotting analysis that revealed that DSG3 overexpression induced phospho-YAP expression as well as total YAP with a significantly increased ratio of p-YAP/YAP compared to control cells. This was coupled with a marked reduction in two *YAP1* target genes, *CYR61/CTGF*, in DSG3 overexpression cells compared to controls, suggesting overexpression of DSG3 reduced YAP nuclear activity [154]. Furthermore, confocal microscopy of phospho-YAP staining in the migrating front of overexpressing DSG3 cells revealed a marked reduction of phospho-YAP nuclear expression and enhanced cytoplasmic distribution as opposed to control cells. Collectively, these findings indicate that DSG3 inhibits YAP nuclear activity by preferentially inducing the expression of inactive phospho-YAP via a complex formation, as reported by our group previously [152,154].

Since DSG3 is a key player in junction assembly, it seems likely that overexpression of DSG3 would enhance cell–cell adhesion strength in H413 cells. However, using the dispase assay that measures cell–cell adhesion strength, we found that DSG3 overexpression in H413 cells did not enhance cell–cell adhesion at all but instead resulted in a trend of cell cohesion weakening compared to control cells [154]. Thus, we argue that DSG3 may function as a key upstream regulator in Hippo signalling to govern the contact inhibition of cell locomotion beyond its characterised role in junction formation and cell adhesion.

To further explore the mechanistic insight, we performed the human phospho-kinase array that revealed that DSG3 suppressed epidermal growth factor receptor (EGFR) signalling in H413 cells. DSG3 overexpression suppressed EGFR S695/Y1086 and its downstream heat shock protein 27 (Hsp27) S78/S82 and transcription factor AP-1 (c-Jun) S63 that trigger YAP activation. Thus, our results indicate that DSG3 inactivates YAP by impeding the EGFR/Hsp27/AP-1/YAP signalling axis in the control of collective migration of OSCC cells [154].

Moreover, an analysis of *YAP1* gene–gene interactions revealed novel Hippo pathway regulators. Although the Hippo downstream effectors YAP/TAZ are well characterised, the upstream regulators remain incompletely elucidated. Our study identified ten DSM genes (*DSG1/3*, *DSC2/3*, *PKP1/2*, *JUP*, *DSP*), in addition to *CDH2* and *CTNNA1*, which are potentially associated with *YAP1* and exhibited a mutually exclusive dependency [154]. However, apart from *DSG3* and *PKP1*, the other DSM genes remain largely unknown in the literature in terms of being linked with *YAP1* function. Thus, our study unravelled almost all DSM components that may potentially play a role in the Hippo pathway to control YAP/TAZ function, similar to α-catenin and E-cadherin-mediated AJs, especially in oral keratinocytes with abundant DSG3 expression [87,90,152,154].

In support, our latest findings found that the desmosomal genes, along with E-cadherin, are the YAP–TEAD transcriptional targets, and that DSG3 regulates key Hippo components, including *WWTR1*/TAZ, *LATS2*, and the other desmosomal molecules [155]. Our latest report indicated that the inhibition of YAP–TEAD interactions by Verteporfin (VP) resulted in a marked reduction of YAP and phospho-YAP and caused a drastic effect on cell–cell junctions, leading to disintegration of both adherens junctions and desmosomes. These findings suggest that YAP expression and the DSG3/phospho-YAP pathway is required for junction formation. Overexpression of DSG3 can rescue, at least in part, VP-mediated damage to cell junctions, resulting in better junction formation and stabilisation [155]. Our study also showed that DSG3 regulates *WWTR1*/TAZ gene expression, with its overexpression leading to elevated *WWTR1* expression [155]. Since the Hippo pathway is intricately regulated depending on the cell context and culture conditions, further investigation is necessary to elucidate how exactly DSG3 regulates TAZ and its activity.

## 6. Conclusions

**Figure 3:**
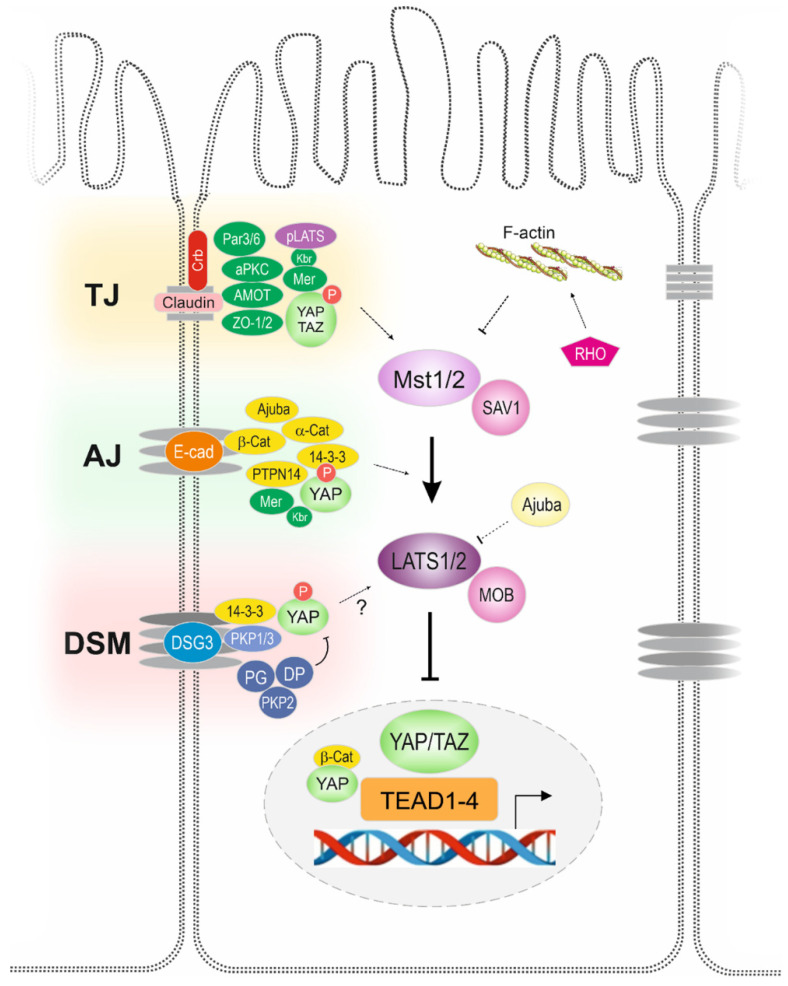
Schematic diagram that summarises cell–cell junctional proteins in the regulation of the Hippo pathway. Several proteins that are associated with tight junctions (TJs), adherens junctions (AJs), and desmosomes (DSMs) have been found to be able to influence the Hippo pathway that, in turn, either activates (Hippo off) or inactivates (Hippo on) YAP/TAZ transcriptional activities in the nucleus. Overall, the majority of these proteins can negatively regulate YAP via directly binding to YAP at the cytoplasmic site of the plasma membrane. However, the desmosomal plaque proteins such as PG (plakoglobin), DP (Desmoplakin), and PKP2 (plakophilin-2) have been found to positively regulate YAP in cardiomyocytes.

YAP/TAZ are tightly regulated proteins that are influenced by multiple intracellular and extracellular stimuli facilitated through components of the Hippo pathway. This review focuses on components of cell–cell junctions, including AJs, TJs, and DSMs, that have been shown to act as upstream regulators of the Hippo pathway (Figure 3). Our recent findings underscore that DSG3 may function as a key component in the Hippo signalling network, as supported by several lines of evidence [152,153,154,155]. Of course, further studies are needed to decipher its role in the Hippo pathway.

## Figures and Tables

**Table 1:** Key Hippo pathway genes in *D. melanogaster* and respective orthologues in humans.

Human Protein Name	Human Gene	*D. melanogaster* Protein (Gene)
Mammalian STE20-like kinase 1 (MST1)	*STK4*	Hippo *(Hpo)*
Mammalian STE20-like kinase 2 (MST2)	*STK3*	Hippo *(Hpo)*
Salvador family WW domain-containing protein 1	*SAV1*	Salvador *(Sav)*
MOB kinase activator 1A	*MOB1A*	Mats *(Mats)*
MOB kinase activator 1B	*MOB1B*	Mats *(Mats)*
Large tumour suppressor kinase 1	*LATS1*	Warts *(Wts)*
Large tumour suppressor kinase 2	*LATS2*	Warts *(Wts)*
Yes-associated protein 1	*YAP1*	Yorkie *(Yki)*
Transcriptional co-activator with PDZ-binding motif	*WWTR1*	Yorkie *(Yki)*
TEA domain family members 1-4	*TEAD1-4*	Scalloped (*Sd*)

## Data Availability

Not applicable.

## Abbreviations

Abbreviations: AJs, adherens junctions; AMOT, angiomotin; aPKC, atypical protein kinase C; c-Jun, transcription factor AP-1; CLDN2, Claudin-2; CLDN4, Claudin-4; CLDN6, Claudin-6; CPE, clostridium perfringens enterotoxin; Crb, Crumbs; CTGF, connective tissue growth factor; CYR61, cysteine-rich angiogenic inducer 61; DIFC, desmosome–intermediate filament complex; Dp, desmoplakin; DSG3, desmoglein-3; DSM, desmosomes; ECM, extracellular matrix; EGFR, epidermal growth factor receptor; EMT, epithelial to mesenchymal transition; ERM, ezrin/radixin/myosin; Ex, expanded; FA, focal adhesion; Hpo, Hippo; Hsp27, heat shock protein 27; LATS1/2, large tumour suppressor kinase 1/2; Kbr, Kibra; Mer, Merlin; MOB1A, MOB kinase activator 1A/B; mRNA, messenger ribonucleic acid; MST1/2, mammalian STE20-like kinase 1/2; NF2, neurofibromatosis type 2; OSCC, oral squamous cell carcinoma; PG, plakoglobin; PKP1, plakophilin-1; PKP2, plakophilin-2; PKP3, plakophilin-3; PTPN14, protein tyrosine phosphatase non-receptor type 14; PV, pemphigus vulgaris; p-YAP, phosphorylated yes-associated protein serine 127; SAV1, Salvador family WW domain-containing protein 1; SCC, squamous cell carcinoma; SCRIB, scribble; TAOK, thousand and one kinase; TAZ, transcriptional co-activator with PDZ-binding motif; TEAD, transcriptional enhanced associate domain; TJs, tight junctions; VP, Verteporfin; WBP2, WW-binding protein 2; YAP, yes-associated protein; ZO, zonula occludens.
